# Long-Term Results of Gamma Knife Radiosurgery for Glomus Tumors: An Analysis of 32 Patients

**DOI:** 10.7759/cureus.18095

**Published:** 2021-09-19

**Authors:** Ryan L Hellinger, Aizik Wolf, Laurie Blach, Lawrence R Kleinberg, Sammie Coy

**Affiliations:** 1 Neurosurgery, Johns Hopkins University, Baltimore, USA; 2 Neurosurgery, Miami Neuroscience Center at Larkin, South Miami, USA; 3 Radiation Oncology, Miami Neuroscience Center at Larkin, South Miami, USA; 4 Radiation Oncology, Johns Hopkins University School of Medicine, Baltimore, USA; 5 Allergy and Immunology, Larkin Community Hospital, Miami Neuroscience Center, South Miami, USA

**Keywords:** glomus tumor, glomus jugulare tumors, brain, brain tumor, brain tumor, gamma knife radiosurgery, stereotactic radiosurgery

## Abstract

Background

Glomus jugulare tumors are rare slow-growing hypervascular tumors that arise from the paraganglia of the chemoreceptor system within the jugulare foramen of the temporal lobe. The historical standard treatment has been surgical resection, but because of their high vascularity and involvement with cranial nerves (CNs), Gamma Knife radiosurgery (GKRS) has been advocated as an alternative. The goal of this study is to update and report long-term results of GKRS to achieve local control and symptomatic improvement and to reduce morbidity and mortality when treating glomus jugulare tumors.

Materials and Methods

This study retrospectively collected and reviewed clinical and radiographic data of 32 patients with glomus jugulare tumors treated with GKRS at the Miami Neuroscience Center, South Miami, FL, from 1995 to 2019. For the 32 patients, the mean volume treated was 13.9 cc (0.23 to 40.0 cc), with an average of 8.6 isocenters. The median prescription dose was 12.84 Gy ± 2.07 Gy (range: 10-20 Gy). Follow-up data were available for 29 out of 32 patients, with a median clinical follow-up time of 37.3 months (range: 4.3-169.1 months). At follow-up, patients were evaluated for neurological signs and symptoms and radiographic evidence of progression of disease.

Results

The median age of the cohort treated with GKRS was 60 years (range: 14-83 years). There were three males and 27 females. Presenting symptomatology was available for 30 out of 32 patients. The most common presenting symptom was hearing loss (21/30) and the most common CN deficit was in CN VIII (19/30). Out of 29 of the patients followed up, 28 patients had improvement (20/29) or resolution (8/29) of symptoms. At the most recent evaluation or contact, patients were without symptomatic progression of CN deficits.

Radiographic tumor control was achieved in 28 out of 29 patients. One patient had a recurrence seven years after GKRS, which was treated with surgery. There were no complications, radionecrosis, or mortality reported from GKRS.

Conclusion

These data confirm that GKRS is a reasonable upfront treatment option for glomus jugulare tumors. GKRS should be considered more frequently given its excellent long-term local control with low morbidity and risk of complications.

## Introduction

Glomus jugulare tumors (GJTs) are rare slow-growing hypervascular benign tumors that arise from the paraganglia of the chemoreceptor system within the jugulare foramen of the temporal lobe [1]. These are the most common tumors of the middle ear and occur predominantly in women, with three to six women for every man diagnosed [2,3]. Common symptoms include hearing loss, pulsatile tinnitus, and lower cranial nerve (CN) deficits [1]. They are multicentric in 3-10% of sporadic cases and 25-50% of familial cases [4]. Due to their slow-growing nature, these lesions are typically diagnosed between the fifth and sixth decade of life [1].

Surgical resection has been the major modality for the treatment of GJTs at diagnosis. Gamma Knife Radiosurgery (GKRS) has historically been reserved for adjuvant treatment or for unresectable or recurrent tumors. Due to their high vascularity and involvement with CNs, GKRS has been advocated as a non-invasive upfront alternative to surgical resection, with favorable results and high local control [1]. The purpose of this study is to update and report long-term results of primary management of GJTs with GKRS to achieve local control and symptom improvement and to reduce morbidity and mortality when treating GJTs.

## Materials and methods

This research was approved by the Institutional Review Board (IRB) at Larkin Hospital (Approval # LCH-7-062021). This study retrospectively collected clinical and radiographic data of 32 patients with GJTs treated with GKRS at the Miami Neuroscience Center, South Miami, FL, from 1995 to 2019. There was a mean volume of 13.9 cc (0.23 to 40.0 cc), with an average of 8.6 isocenters. The mean treatment time was 1 hour and 6 minutes (range: 9 minutes to 2 hours and 59 minutes). One treatment was staged as a 56-minute treatment of the superior 22.2-cc portion and a 17-minute treatment of the more difficult to reach 8.3-cc inferior portion.

The Leksell Gamma Knife® treatment was carried out using the Model U from 1995 to 2000, the Model C from 2000 to 2008, and the Perfexion Unit from 2008 to present (Elekta Instruments, Stockholm, Sweden). The Leksell Gamma Knife stereotactic frame was placed on each patient’s head with quick pins using lidocaine as local anesthesia after premedication with 1 g of cefazolin and 20 mg of dexamethasone (intravenous sedation with propofol and midazolam was introduced in the last decade). Stereotactic localization of the lesion, visualization, and volume dosimetry mapping were based on MRI. Radiosurgical treatment planning was performed in conjugation with a neurosurgeon, radiation oncologist, and medical physicist using gamma plan. The mean prescription dose was 12.84 Gy ± 2.07 Gy (range: 10-20 Gy). The prescription dose was 50% of the maximum dose in 70% of the treatments, ranging from 40% to 85%.

Follow-up consisted of brain surveillance MRI and clinical neurological evaluation at one month, three months, and then regularly every six months after GKRS or if there were new or worsening symptoms. Three out of 32 patients were lost to follow-up. The median clinical follow-up time was 37.3 months (range: 4.3-169.1 months). Patients were evaluated for neurological and radiological progression of disease at every follow-up. Clinical status was assessed by a complete neurological exam and patient history, whereas radiographic status was determined by measuring tumor volume in cubic centimeters via MRI imaging. Neurological and radiographic progression was then determined to be stable, improved or worsened based on the change in pretreatment to post-treatment status. Symptomatology and tumor volume were categorized as stable if there were no changes in symptoms or tumor volume at follow-up compared to at GKRS. Symptoms were classified as improved if there was a relief of symptoms and worsened if there was an aggravation in symptoms. Radiographic progression was labeled as improved if there was a decrease in tumor volume and worsened if there was an increase in tumor volume.

## Results

The median age of our patient cohort was 60 years (range: 14-83 years). There were three males and 27 females. Eleven patients underwent initial treatment prior to GKRS: eight with surgical resection, two with external beam radiation therapy (EBRT), and one with EBRT, surgery, and initial stereotactic radiosurgery (SRS). These 11 patients were referred to our institution due to progression of tumor growth and/or symptoms after their initial treatment.

Presenting symptomatology was available in 30 out of 32 patients. The tumors were left sided in 15 patients. Patients presented with hearing loss (21/30) and/or tinnitus (20/30) in 28 out of 30 cases, making hearing difficulties the most common symptom (Table 1). Other common symptoms included dysphagia (14/30), headaches (13/30), vision problems (11/30), and imbalance (11/30) (Table 1). The most common neurological deficit was CN VIII in 19 patients, whereas other CN deficits were present in CNs IX, XII, VII, X, and XI (Table 2). Three patients had no CN deficits prior to treatment.

**Table 1:** Patient characteristics *Presenting symptoms were available in 30 out of 32 patients.

Patient Parameter	Number of Cases (%)
Median age	60
Sex	
Male	3 (10.0%)
Female	27 (90.0%)
Tumor location	
Left hemisphere	15 (50%)
Right hemisphere	15 (50%)
Presenting symptoms*	
Hearing loss	21 (70.0%)
Tinnitus	20 (66.7%)
Dysphagia	14 (46.7%)
Headaches	13 (43.3%)
Vision problems	11(36.7%)
Imbalance	11 (36.7%)

**Table 2:** Cranial nerve deficits

Cranial Nerve Deficits	Number of Cases (%)
V	3 (10.0%)
VI	2 (6.67%)
VII	8 (26.7%)
VIII	19 (63.3%)
IX	12 (40.0%)
X	9 (30.0%)
XI	9 (30.0%)
XII	10 (33.3%)
None	3 (10.0%)

**Figure 1:**
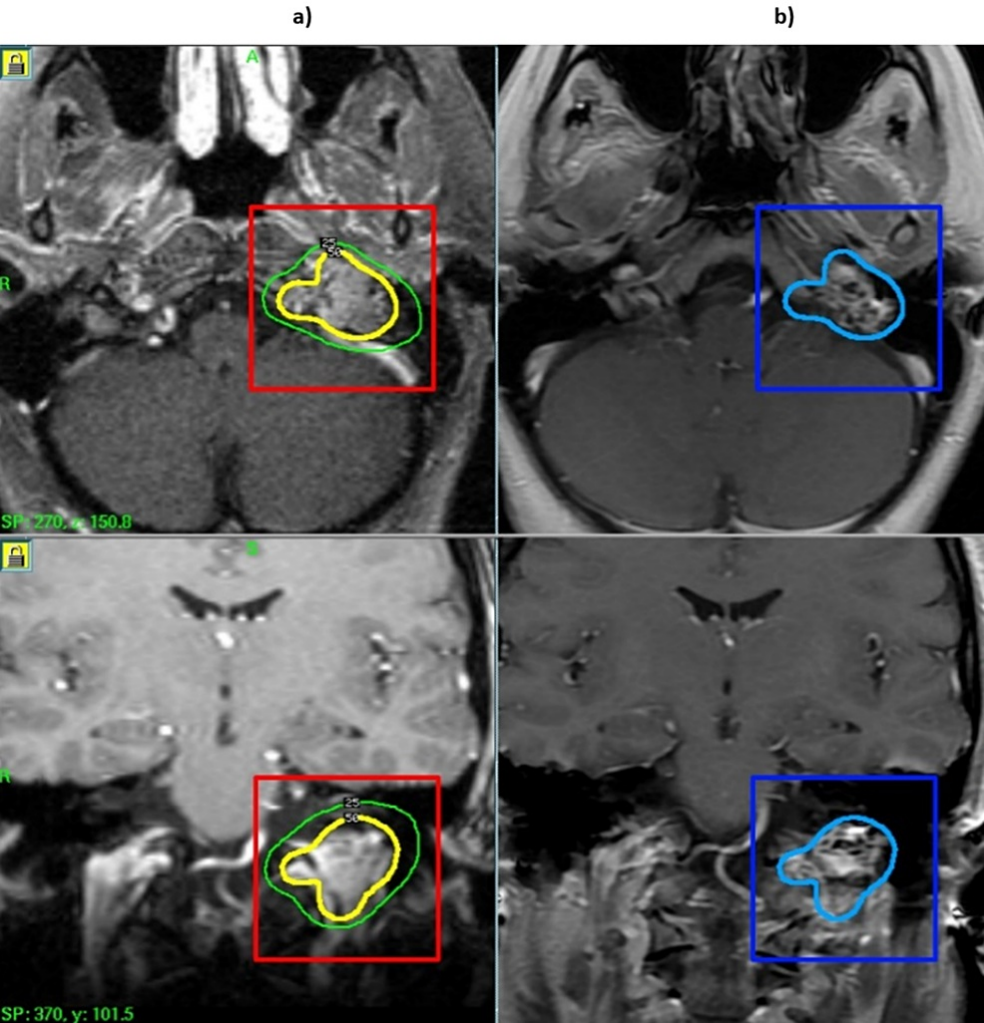
MRI of glomus jugulare tumor before and after GKRS (a) Pre-GKRS MRI of a patient with a 6.7-cc glomus tumor treated with 12 Gy to the 50% isodose line. (b) Post-GKRS MRI of the same patient five years after GKRS showing tumor shrinkage. GKRS, Gamma knife radiosurgery

The median follow-up time of the 29 out of 32 patients was 37.3 months. Out of the 29 patients, five had over five years of follow-up. Tumor control, defined as the absence of radiographic or clinical evidence of progression of tumor growth, was achieved in 28 out of 29 patients at any point after GKRS. Decrease in tumor size occurred in 11 (39%) out of 29 patients, and 17 (61%) out of 29 had tumor stabilization post-GKRS (Figure 1). One patient had a recurrence resulting in surgery seven years after GKRS (1/29).

**Figure 2:**
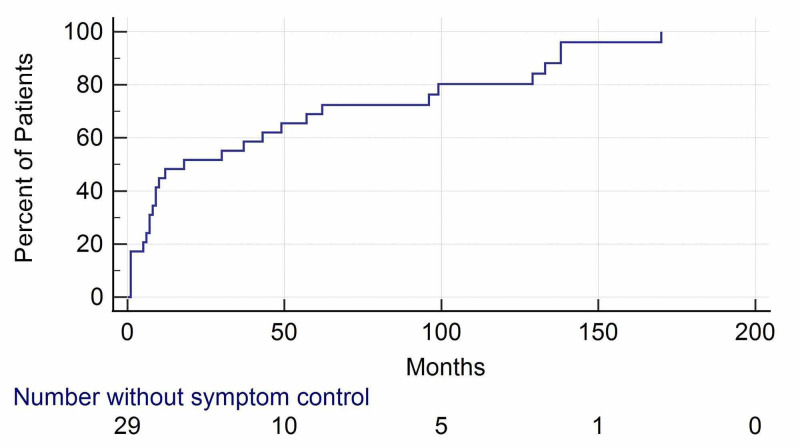
Kaplan-Meier curve illustrating time to symptom control of the 29 patients followed up Symptom control is defined as stable or improved symptoms.

Of the 29 patients followed up, 28 responded favorably with improved (20/29) or resolved (8/29) symptoms after GKRS. Only the one patient (1/29) who required surgery seven years after GKRS had unresolved symptoms. After one month, one patient had their hearing issues partially controlled post-GKRS, with the rest having hearing issues completely resolved. Headaches were resolved in 12 out of 13 cases. Dysphagia and vision problems were ameliorated in all cases. CN deficits were stabilized in all cases. The majority of symptom control, defined as stable or improved symptoms, occurred within the first 50 months of follow-up (Figure 2). At the most recent follow-up, no patients developed new or progressive symptomatology. The 11 patients who received prior treatment had all their symptoms either stabilized or resolved after GKRS at this center. There were no complications, radionecrosis, or mortality reported from GKRS.

## Discussion

This study adds to the body of literature supporting the use of GKRS alone as an upfront definitive management of GJTs [1-2,5-27]. This analysis confirms that GKRS effectively treats GJTs with excellent long-term tumor control. The hypervascularity of glomus tumors makes it a surgical challenge, and while surgical resection may be simple for smaller tumors, large tumors that affect the lower CNs and extend beyond the petrous apex carry a significant risk of postoperative complications. Furthermore, surgery may be contraindicated based on age and general physical condition of the patient. Therefore, the non-invasive option of GKRS may be the preferred treatment.

The results of this study are consistent with the results of other glomus jugulare GKRS studies, including the study by Hafez et al. [28]. They report similar patient characteristics including sex and age. As in our study, the majority received GKRS as primary treatment (15/22), with the remaining receiving GKRS as adjuvant therapy. Unlike our study, the patient cohort sustained CN deficits most commonly in CNs IX, X, and XI. They used a slightly higher mean marginal dose of 14.7 Gy compared to our dose of 12.84 Gy. Resembling our study, all cases in this study had tumor control or reduction in size. However, there were complications in three (13.6%) cases. One developed trigeminal neuralgia, one facial nerve palsy, and one hearing loss and facial numbness [28].

A comparable paper, Wakefield et al. [2], collected data from 17 patients who received GKRS for GJTs. There was a nearly equal distribution of males and females (seven males, 10 females), with a median age of 64 years. This is inconsistent with our study and that by Hafez et al., which reported a much higher proportion of females than males [28]. Like our study, the most common symptom was hearing loss (11/17). This study also used a slightly higher median marginal dose of 15 Gy. Overall, this study’s results coincided with ours with all patients, except for one, experiencing improved (53%) or stabilized (41%) symptoms. Furthermore, all patients, except one, experienced tumor shrinkage (59%) or stabilization (35%). Also, only one patient endured a recurrence five years after surgery. This study had no operative complications or mortality [2].

A more recent study by Tse et al. [29] collected data from 13 patients who received SRS between 2009 and 2016 for glomus tumors. There were 13 females and 0 males, with a median age of 63 years. The most common presenting symptoms were hearing loss and pulsatile tinnitus, consistent with our study. They delivered a much higher prescribed dose of 27.6 Gy compared to our institution and the previous studies, [2,28-29]. Consistent with our results, tumor control occurred in 92.3% of patients, with a median follow-up of 47.4 months, and only one patient experienced tumor recurrence (Table 3). The majority of patients achieved resolution of tinnitus (87.5%). Complications were reported in three patients: one patient developed transient facial nerve palsy, one trigeminal neuralgia, and one hearing loss [29].

**Table 3:** Comparison of GJTs treatment options among five studies GKRS, Gamma Knife radiosurgery; STR, subtotal resection; gross total resection; SRS, stereotactic radiosurgery; GJT, glomus jugulare tumor *One patient had surgery seven years after GKRS

Study	Treatment	Number of Cases	Tumor Control	Median Follow-Up (Months)
This study	GKRS	30	96.55%*	37.3
Hafez et al. [28]	GKRS	22	100%	-
Wakefield et al. [2]	GKRS	17	94.12%	123
Tse et al. [29]	GKRS	13	92.3%	47.4
Ivan et al. [30]	STR	869	69%	72
	GTR		86%	88
	STR + SRS		71%	96
	SRS		95%	71

GKRS appears to potentially achieve a higher local control with fewer complications compared to standard surgical resection. Ivan et al. [30] analyzed data of 869 patients with GJTs from 109 studies and compared the rates of recurrence and neuropathy between four different treatment approaches including gross total resection (GTR), subtotal resection (STR) alone, STR with adjuvant postoperative radiosurgery (STR + SRS), and SRS alone. Tumor control was superior in 86% of cases treated with GTR versus 95% tumor control in SRS, 69% in STR, and 71% in STR+SRS [30]. Furthermore, this analysis encountered higher complication rates after GTR and worse postoperative CN deficits in CNs IX-XI compared to SRS alone. Of these studies, SRS alone possessed the highest tumor control with the lowest CN deficits postoperatively, making it the most favorable form of treatment (Table 3).

The effectiveness of GKRS on GJTs compared to surgery is illustrated by the cohort of patients from our study who underwent initial resection and then GKRS several years after surgery due to recurrence. GKRS treated the tumors in all cases where surgery failed to do so. Even the combination of resection with adjuvant radiation therapy approach, previously touted as the best option, failed to control the tumor as well as GKRS alone [30] (Table 3). Comparison of GKRS to alternative treatments illustrates that GKRS results in lower rates of morbidity and mortality. This supports the claim that GKRS for GJTs is a safer and more effective treatment contrasted with its alternatives.

There are limitations in this case series due to its retrospective nature and lack of homogenous data. We acknowledge that it is difficult to conclude the independent effects of GKRS on GJTs because some patients sustained prior treatment to the GJT. Furthermore, some patients were international; therefore, there is a lack of detail in follow-up data in some cases, including the patient with a recurrence seven years after GKRS. To correct for this, follow-up variables were standardized to stable, worsened, or improved symptomatic and tumor control. We recognize that it is difficult to make recommendations for clinical practice due to the lack of a reference group. Prospective studies comparing GKRS to another treatment method should be employed to make a more definitive recommendation on the use of GKRS but would be unlikely due to the low incidence of GJTs.

## Conclusions

Our data confirm that GKRS is a reasonable upfront treatment of GJTs given the high long-term local control without significant risk of complications. GKRS can be offered as an upfront alternative treatment of GJTs in unresectable patients or patients with high surgical risk as local control is maintained and the morbidity profile of this approach remains low.
